# Analysis of clogging factors in single-wing labyrinth drip irrigation tape

**DOI:** 10.1371/journal.pone.0313888

**Published:** 2024-12-31

**Authors:** Hui Zhang, Hongfei Tao, Yao Liu, Qiao Li, Youwei Jiang, Mahemujiang Aihemaiti, Wenxin Yang

**Affiliations:** 1 College of Hydraulic and Civil Engineering, Xinjiang Agricultural University, Urumqi, China; 2 Xinjiang Key Laboratory of Hydraulic Engineering Security and Water Disasters Prevention, Urumqi, China; Ardakan University, ISLAMIC REPUBLIC OF IRAN

## Abstract

The clogging performance of single-winged labyrinth drip irrigation tapes is influenced by a variety of factors during the muddy fertilizer water irrigation process. In this paper, we designed a uniform orthogonal test to study the effects of fertilizer concentration, sediment content and working pressure on the clogging of single-wing labyrinth drip irrigation tapes. The observed data from the experiment were analysed and calculated using range analysis, variance analysis, and main-effect multiple comparison analysis, then the optimal working conditions were determined. The results of the study showed that fertilizer concentration and sediment content had a significant effect on the average relative flow, and the degree of influence of each influencing factor on the average relative flow was as follows: sediment content > fertilizer concentration > working pressure. The average relative flow of water-fertilizer-integrated irrigation under muddy-water conditions decreased with the increasing of irrigation frequency, and accelerate the clogging of dripper. Eventually we concluded that muddy fertilizer water irrigation exacerbated the clogging of dripper. The highest average relative flows across three sediment levels occurred under the F1 treatment, measuring 0.699, 0.681, and 0.668 for type-H_1_, type-H_2_, and type-H_3_ tapes, respectively. The research results can provide a reference basis for the optimal design of structural parameters of single-wing labyrinth drip irrigation tape channel.

## 1. Introduction

Water scarcity is a critical challenge for global agriculture, especially in arid and semi-arid regions, where it limits economic growth and crop production. Drip irrigation, recognized for its water efficiency and precision in nutrient delivery, has become an essential technology in addressing these challenges [1, 2]. However, clogging of drip irrigation systems, often caused by sediment content and particle size, remains a significant issue that adversely affects system performance and longevity [3, 4]. Factors such as laying slope, working pressure, and manufacturing deviations further influence the hydraulic performance and susceptibility to clogging of the drip irrigation tapes [5, 6].

Dripper clogging has always been a focal point of research [7, 8]. Preventing dripper clogging of drip irrigation tape and improving irrigation efficiency are the key issues of their research focus. Currently, the main methods for clogging treatment include pretreatment and timely cleaning of the drip irrigation tapes and filters [9]. The clogging of drip irrigation tapes can be roughly classified as physical clogging, chemical clogging, and biological clogging. Nakayama also assessed the water quality due to clogging of dripper and proposed a water quality classification table for clogging of irrigators [7]. Water quality is the most fundamental cause of dripper clogging, and it has a significant influence on the hydraulic performance of the drip irrigation tape. Adin et al. conducted a drip irrigation experiment with sewage water to study the clogging of the dripper and found that the form of the flow channel of the dripper had a significant influence on the clogging performance [10]. Xu et al. verified the numerical simulation results by using short-period scale inhibition experiments, and revealed the relationship between the hydraulic performance and anti-clogging performance of the dripper [11]. Qiu et al. used physical experiments and numerical simulations to explore the effects of different tooth shape designs on dripper flow characteristics and anti-clogging performance, and pointed out that the BUV (teeth are perpendicular to the upstream vertical surface) flow channel has the best improvement effect on the anti-clogging performance of the dripper [12]. Zhou et al. used three kinds of inferior water to carry out drip irrigation tape clogging test to determine the anti-clogging ability of different drippers, and determined the parameters that can be directly used to evaluate the anti-clogging ability of drippers [13]. Wang et al conducted an in-situ field study to investigate the impacts of saline water concentrations and water-soluble P-fertilizer types on DI system performance, and explored the dynamic variation of chemical precipitations accumulated in drip irrigation drippers [14]. Zhang et al. selected four different water salinities to conduct an on-site drip irrigation experiment in Hetao Irrigation District, and analysed the behavior and dripper distribution of chemical clogging in a drip irrigation system [15]. Li et al. conducted a drip irrigation experiment with four irrigation and flushing treatments to study the chemical precipitates dynamic variations in the clogging substances, and reveal the mechanism of chemical precipitates and their impacts on the clogging process [16].

Under muddy-water conditions, the form of the flow channel exerts a significant influence on dripper clogging. Additionally, factors such as fertilizer concentration, sediment content, and working pressure also play crucial roles in this process. Although the current water and fertilizer integrated drip irrigation technology is gradually being popularized, the problem of dripper clogging has been further aggravated. Li et al. demonstrated that fertilization significantly exacerbates dripper clogging by inducing the aggregation of sediment particles [17]. Wang et al. conducted a study and found that large amounts of carbonate sediments and fertilizers are the most important reasons for the severe clogging of drip irrigation systems [18]. Liu et al. found that different types of fertilizers have significant influences on the clogging of the dripper [19].

Dripper clogging is a multifaceted problem influenced by various factors, yet most studies have focused on single-factor effects. This study addresses the gap by employing a uniform orthogonal test to examine the combined impact of fertilizer concentration, sediment content, and working pressure on the average relative flow in single-winged labyrinth drip irrigation tapes. Through range analysis, variance analysis, and multiple analysis of main effects, we ranked the significance of these factors and explored their interactions. Our findings establish critical relationships between clogging factors and flow performance, providing essential insights for optimizing water-fertilizer integrated irrigation systems.

## 2. Materials and methods

### 2.1. Test materials and equipment

**Fig 1:**
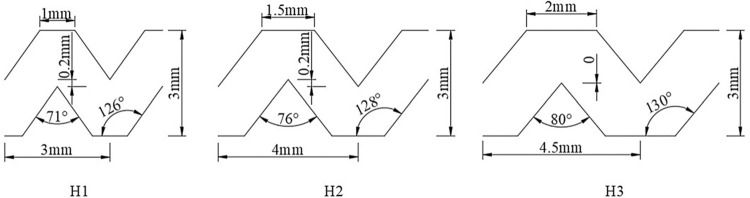
Photograph of drip irrigation tape.

In this study, the single-wing labyrinth drip irrigation tapes with flows of 1.8, 2.6, and 3.2 L/h produced by Xinjiang Tianye Water Saving Irrigation Co., Ltd. were selected. The hydraulic performance parameters of the drip irrigation tapes are shown in Table 1. The size parameters of these tapes are shown in Fig 1.

**Table 1:** Hydraulic performance parameters of the drip irrigation tapes.

Type	Outer diameter (mm)	Outlet distance (cm)	Number of teeth in the trapezoidal channel	Nominal pressure (kPa)	Rated flow (L/h)	Flow coefficient	Flow index
H_1_	16	30	85	100	1.8	0.11	0.60
H_2_	16	30	63	100	2.6	0.16	0.61
H_3_	16	30	54	100	3.2	0.25	0.55

Note: Outer diameter: the diameter of the outer circumference of the cross-section of the drip irrigation tape; Outlet distance: the distance between two adjacent drippers on a drip irrigation tape.

**Fig 2:**
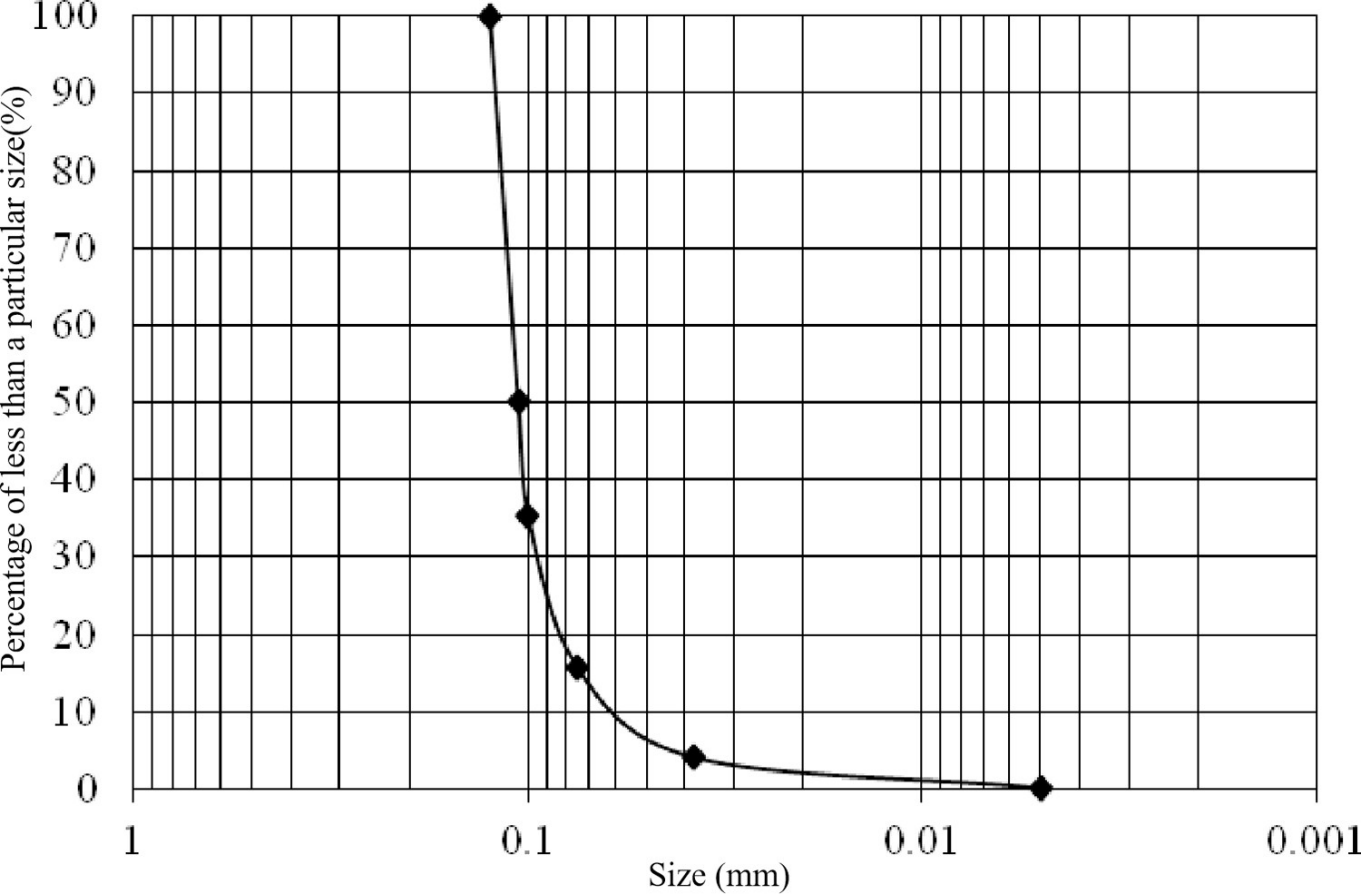
Sediment particle size curve.

The fertilizer used in the experiment was manufactured by Jiashili(Yingcheng) Chemical Fertilizer Co., Ltd. The most commonly used potassium-sulphate-type compound fertilizer was chosen, which has high water solubility, easy absorption characteristics, high nutrient contents, few auxiliary components, and with good physical properties. The proportion of nutrients N:P_2_O_5_:K_2_O was 1: 1: 1, and the total nutrient content was ≥51%. The silt was natural loess from Xishan Mountain in Urumqi, and it was sieved through using a 120-mesh sieve. First, a set of standard sieves was used to sieve the particles with diameters larger than 0.074 mm, the material in each sieve was collected, and the particles were weighed to obtain the percentage of the soil weight. If the particle size was less than 0.074 mm, a certain amount of soil suspension with a uniform concentration was prepared with a graduated cylinder. The suspension densities at different times were measured, and the weight percentage of particles in the soil was calculated based on the densitometer reading and the soil particle settling time. Finally, the distribution curve of the particle size was obtained, as shown in Fig 2. It can be seen that 100% of the particles had particle sizes smaller than 0.125 mm, 35.28% were smaller than 0.1 mm, and the median particle size (D50) was 0.106 mm.

**Fig 3:**
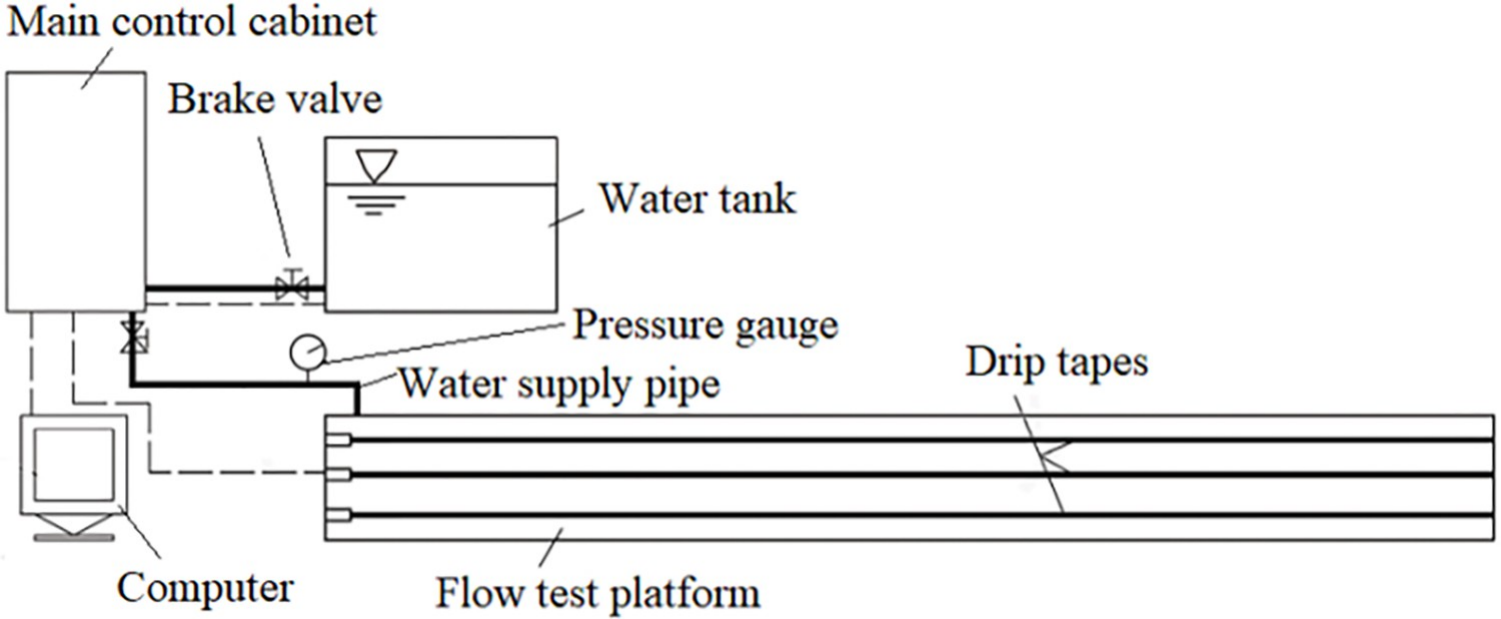
Schematic diagram of the test platform for anti-clogging performance of drip irrigation tape (pipe).

The schematic diagram of the device is shown in Fig 3. This drip irrigation tape anti-clogging performance test bench was a KD-DJC, which was manufactured by Hebei Kedao Testing Machine Technology Co., Ltd., and the system was suitable for a voltage of 380 V. The main control cabinet included a Xilin SD 200 vector inverter. The frequency range of the device is between 0–600 Hz, the load frequency range was between 2–10 kHz, and the speed regulation range was 1:50 or 1 Hz/150% rated torque. A 32 CDLF4-150 light multi-stage pump, produced by Yongjia Yingke Pump Valve Co., Ltd., was used for water injection. The flow was 4 m^3^/h, the pump speed was 2880 r/min, the lift was 120 m, and the power was 3 kW. A YE2-802-2 three-phase asynchronous motor was used to provide 11 kW of powder. The voltage was 380 V, the frequency was 50 Hz, and the rotation speed was 2830 r/min. An IRK 50–100 centrifugal pump was used with a flow of 22.3 m^3^/h, lift of 10 m, matching power of 1.1 kW, and rotation speed of 2900 r/min. The electronic balance model used in this study is the YP2002 N, manufactured by Shanghai Jinghai Instrument Co., Ltd., with a maximum range of 2000 g and an accuracy of 0.01 g.

The length of the drip irrigation tape test platform is 35 m, and a total of 3 drip irrigation tapes are laid. Each drip irrigation tape is equipped with 25 water collection buckets, with total of 75 water collection buckets. The dripper flow was measured by weighing method. Measurements were made every 15 minutes, and record the average of three readings per measurement.

In the test, under the premise of fixed laying length (35m) and slope (0%), the three factors of sediment content, fertilizer concentration and working pressure were used as the selection factors. Three levels were selected for each factor, and the specific factor levels were shown in Table 2. The uniform orthogonal design table UL_9_ (3^3^) was used to design the test scheme. The specific design is shown in Table 3.

**Table 2:** Factors and levels.

Factor	Level		
	1	2	3
Fertilizer concentration(g/L)	1.0	2.0	3.0
Sediment content(g/L)	0.6	1.8	3.0
Working pressure(kPa)	40	70	100

**Table 3:** Uniform orthogonal test UL_9_(3^3^).

	Factor level		
	Fertilizer concentration (g/L)	Sediment content (g/L)	Working pressure (kPa)
1	0.6	1	40
2	0.6	2	100
3	0.6	3	70
4	1.8	1	100
5	1.8	2	70
6	1.8	3	40
7	3	1	70
8	3	2	40
9	3	3	100

To expedite the experiment and broaden the model’s prediction interval, we selected irrigation water with suspended solids concentrations exceeding the farmland irrigation water quality standard by more than tenfold, by using a mass concentration of ≤ 0.1 g/L as the baseline. The sediment concentrations were set at 1, 2, and 3 g/L. To better simulate actual irrigation conditions and ensure that sediment particles fully collide, coagulate, and deposit within the flow channels, both the irrigation duration and intervals were extended within feasible limits. Irrigation was conducted in 10-minute cycles with 30-minute intervals. A total of 9 irrigation cycles were conducted, with flows measured after each cycle. After each treatment, new drip irrigation tapes were installed, and the system components, including pipes, water tanks, and pumps, were thoroughly flushed.

### 2.2. Evaluation index

In this test, the average relative flow was used as the evaluation index to assess clogging in the drip irrigation tape. Clogging was determined based on whether the average relative flow fell below 0.75 (GB/T19812.3–2017). The formula of the average relative flow is as follows:

$$q=\frac{\sum_{1}^{N}\frac{q_{pi}}{q_{i}}}{N},$$ (1)

where *q* is the average relative flow, *i* is the drop number, *N* is the total number of drops, *q*_pi_ is the muddy-water flow of the *i*^th^ dripper (L/h), and *q*_i_ is the water flow of the *i*^th^ dripper (L/h).

## 3. Results and analysis

Combined with the data, the effects of working pressure (B), sediment content (F) and fertilizer concentration (E) on the average relative flows under the condition of drip irrigation with muddy-water (irrigation tape length is 35 m, slope is 0%) were analysed. The influences of these factors on the average relative flow of the single-wing labyrinth drip irrigation tapes were examined, the order of the influence of these factors on the average relative flow was determined, and the relationships between the factors affecting the clogging and the average relative flow were established. The relationship between the relative flow and the optimal operating conditions in this case were explored.

### 3.1. Analyse the test results under muddy-water conditions using SPSS software

The range analysis and multiple comparison analysis of the main effects were carried out on the measurements under muddy-water to explore the relationship between each factor and the test index, then to determine the effects of the working pressure (B), sediment content (F), and fertilizer concentration (E). The order of importance of the influence factors on the average relative flow of the single-wing labyrinth drip irrigation tape was determined.

### 3.2. Test results

The tests were carried out based on an orthogonal experiments scheme, and the average relative flow under muddy-water condition was calculated using formula (1). The results are shown in Table 4.

**Table 4:** UL_9_(3^3^) uniform orthogonal design and test results.

	Factor level			Average relative flow (*q*)		
	E	F	B			
	Fertilizer concentration	Sediment content	Working pressure	H_1_	H_2_	H_3_
	(g/L)	(g/L)	(kPa)			
1	0.6	1	40	0.793	0.702	0.764
2	0.6	2	100	0.653	0.628	0.653
3	0.6	3	70	0.564	0.523	0.587
4	1.8	1	100	0.702	0.693	0.711
5	1.8	2	70	0.564	0.657	0.588
6	1.8	3	40	0.523	0.502	0.534
7	3	1	70	0.603	0.647	0.621
8	3	2	40	0.55	0.573	0.548
9	3	3	100	0.431	0.457	0.451

### 3.3. Range analysis

Tables 5, 6 and 7 show the range analysis results of the average relative flow for different values of the test factors in the H_1_, H_2_, and H_3_ single-wing labyrinth drip irrigation tapes. The order of the influence factors (laying pressure (B), sediment content (F), and fertilizer concentration (E)) on the average relative flow of the single-wing labyrinth drip irrigation tape under muddy-water conditions for the H_1_, H_2_, and H_3_ single-wing labyrinth drip irrigation tapes was as follows: R_F_ > R_E_ > R_B_ > R_D_. The order of the factors affecting the average relative flow of the drip irrigation tape was as follows: F(sediment content) > E(fertilizer concentration) > B(working pressure).

**Table 5:** Results of the range analysis of each factor (H_1_).

Group number	Factor level				Average relative flow (q)
	E	F	B	D	
	Fertilizer concentration (g/L)	Sediment content (g/L)	Working pressure (kPa)		
1	0.6	1	40	2	0.793
2	0.6	2	100	1	0.653
3	0.6	3	70	3	0.564
4	1.8	1	100	3	0.702
5	1.8	2	70	2	0.564
6	1.8	3	40	1	0.523
7	3	1	70	1	0.603
8	3	2	40	3	0.55
9	3	3	100	2	0.431
K_1j_	2.01	2.098	1.866	1.779	
K_2j_	1.789	1.767	1.731	1.788	
K_3j_	1.584	1.518	1.786	1.816	
k_1j_	0.670	0.699	0.622	0.593	
k_2j_	0.596	0.589	0.577	0.596	
k_3j_	0.528	0.506	0.595	0.605	
R_j_	0.142	0.193	0.045	0.012	
Order of R	R_F_>R_E_>R_B_>R_D_				

Note: R_i_ represents the range of the average relative flow of different types of drip irrigation tapes. D: the error in range analysis.

**Table 6:** Results of the range analysis of each factor (H_2_).

Group number	Factor level				Average relative flow (q)
	E	F	B	D	
	Fertilizer concentration (g/L)	Sediment content (g/L)	Working pressure (kPa)		
1	0.6	1	40	2	0.702
2	0.6	2	100	1	0.628
3	0.6	3	70	3	0.523
4	1.8	1	100	3	0.693
5	1.8	2	70	2	0.657
6	1.8	3	40	1	0.502
7	3	1	70	1	0.647
8	3	2	40	3	0.573
9	3	3	100	2	0.457
K_1j_	1.853	2.042	1.777	1.777	
K_2j_	1.852	1.858	1.827	1.816	
K_3j_	1.677	1.482	1.778	1.789	
k_1j_	0.618	0.681	0.592	0.592	
k_2j_	0.617	0.619	0.609	0.605	
k_3j_	0.559	0.494	0.593	0.596	
R_j_	0.059	0.187	0.017	0.013	
Order of R	R_F_>R_E_>R_B_>R_D_				

Note: R_i_ represents the range of the average relative flow of different types of drip irrigation tapes. D: the error in range analysis.

**Table 7:** Results of the range analysis of each factor (H_3_).

Group number	Factor level				Average relative flow (q)
	E	F	B	D	
	Fertilizer concentration (g/L)	Sediment content (g/L)	Working pressure (kPa)		
1	0.6	1	40	2	0.764
2	0.6	2	100	1	0.653
3	0.6	3	70	3	0.587
4	1.8	1	100	3	0.711
5	1.8	2	70	2	0.588
6	1.8	3	40	1	0.534
7	3	1	70	1	0.621
8	3	2	40	3	0.548
9	3	3	100	2	0.451
K_1j_	2.004	2.096	1.846	1.808	
K_2j_	1.833	1.789	1.796	1.803	
K_3j_	1.62	1.572	1.815	1.846	
k_1j_	0.668	0.699	0.615	0.603	
k_2j_	0.611	0.596	0.599	0.601	
k_3j_	0.540	0.524	0.605	0.615	
R_j_	0.128	0.175	0.017	0.014	
Order of R	R_F_>R_E_>R_B_>R_D_				

Note: R_i_ represents the range of the average relative flow of different types of drip irrigation tapes. D: the error in range analysis

**Fig 4:**
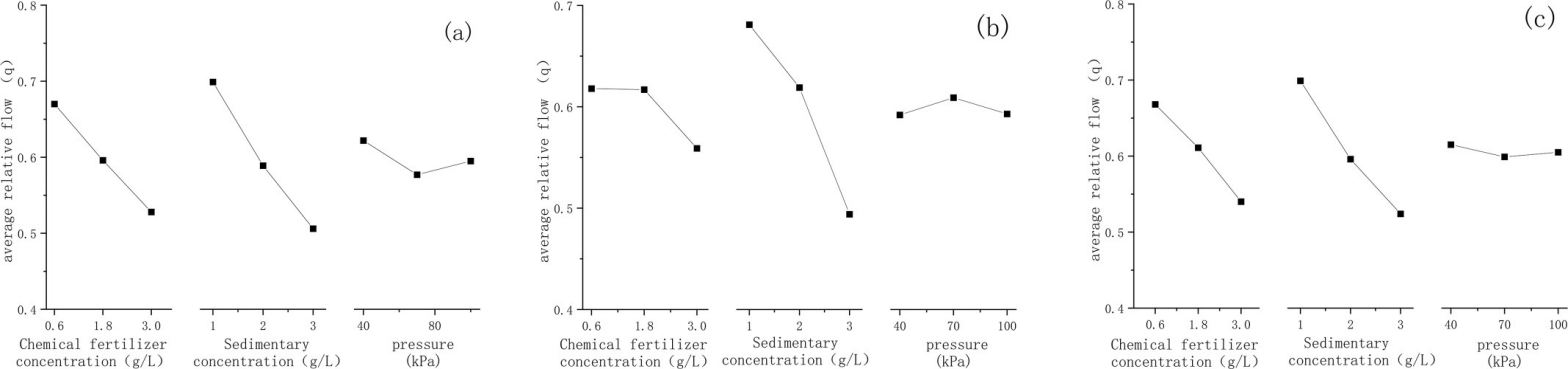
Average relative flow of drip irrigation tape with different factor values: (a) drip irrigation tape type H_1_, (b) drip irrigation tape type H_2_, and (c) drip irrigation tape type H_3_.

The relevant factor indicators are shown in Fig 4. As shown in Fig 4(A), the optimal average relative flow occurred when the fertilizer concentration is 0.6 g/L, sediment content is 1 g/L, and working pressure is 40 kPa in the type-H_1_ single-wing labyrinth drip irrigation tape. As for Fig 4(B), the optimal average relative flow occurred when the fertilizer concentration is 0.6 g/L, sediment content is 1g/L, and working pressure is 40 kPa in the type-H_2_ single-wing labyrinth drip irrigation tape. As for Fig 4(C), the optimal average relative flow occurred when the fertilizer concentration is 0.6 g/L, sediment content is 1g/L, and working pressure is 70 kPa in the type-H_3_ single-wing labyrinth drip irrigation tape.

### 3.4. Variance analysis and multiple comparison analysis of principal effects

#### 3.4.1. Variance analysis

The results of the variance analysis are shown in Table 8. At the 95% probability level, the results showed that the fertilizer concentration and sediment concentration of the type-H_1_ drip irrigation tape reached the extremely significant level (p < 0.01). For the type-H_2_ and type-H_3_ drip irrigation tapes, the sediment concentration reached the extremely significant level, and the fertilizer concentration reached the significant level (p < 0.05). The effects of the working pressure on the relative flows of the three drip irrigation tapes did not reach the significant level. This showed that the fertilizer concentration and the sediment concentration had significant impacts on the relative flows of the drip irrigation tapes, and thus, they would be important factors causing the dripper to clog. The fertilizer concentration of the type-H_1_ drip irrigation tape reached the extremely significant level, while for the type-H_2_ and type-H_3_ drip irrigation tapes, it only reached the significant level. All three types of drip irrigation tapes were single-wing labyrinth flow channels, but the type-H_1_ drip irrigation tape was relatively narrower than the type-H_2_ and type-H_3_ drip irrigation tapes. To a certain extent, this narrow tape enhanced the collision and flocculation of sediment particles after fertilization, making it easier to form a stable agglomeration mechanism to clog the dripper. From the F values, it can be seen that the order of the factors affecting the average relative flow of the three single-wing labyrinth drip irrigation tapes was F > E > B, that is, sediment content > fertilizer concentration > working pressure, and the variance analysis results were consistent with the range analysis results.

**Table 8:** Variance analysis of average relative flow.

Drip irrigation tape	Variance source	Quadratic sum	Degrees of freedom	Mean square	F value	P value
H_1_	Fertilizer concentration	0.030	2	0.015	121.908	0.008
	Sediment concentration	0.056	2	0.028	227.378	0.004
	Working pressure	0.003	2	0.002	12.377	0.075
	Error	0.000	2	0.000		
H2	Fertilizer concentration	0.007	2	0.003	25.732	0.037
	Sediment concentration	0.054	2	0.027	204.190	0.005
	Working pressure	0.001	2	0.000	2.048	0.328
	Error	0.0003	2	0.0001		
H3	Fertilizer concentration	0.025	2	0.012	66.928	0.015
	Sediment concentration	0.046	2	0.023	125.351	0.008
	Working pressure	0.000	2	0.000	1.152	0.465
	Error	0.0004	2	0.0002		

#### 3.4.2. Multiple comparison analysis of principal effects

SPSS 22.0 was used to analyse the differences between different levels of the influence factors. The range analysis showed that the influence of the working pressure (B) was not significant, so multiple comparison analysis regarding to working pressure (B) was not conducted. The multiple comparison results of the principal effects are shown in Table 9.

**Table 9:** Multiple comparison analysis of principal effects.

Drip irrigation tapes	Factors	Average relative flow			
		Significance	Level	Average	Duncan
					α = 0.05
H_1_	B	0.075	-	-	-
	E	0.008**	2	0.596	a
			3	0.528	b
			1	0.670	c
	F	0.004**	1	0.699	a
			2	0.589	b
			3	0.506	c
H_2_	B	0.328	-	-	-
	E	0.037*	1	0.618	a
			2	0.599	b
			3	0.617	b
	F	0.005**	1	0.681	a
			2	0.619	b
			3	0.494	c
H_3_	B	0.465	-	-	-
	E	0.015*	1	0.668	a
			2	0.611	b
			3	0.540	c
	F	0.008**	1	0.698	a
			2	0.596	b
			3	0.524	c

The comparison of fertilizer concentration (E) and sediment content (F) across different drip irrigation tapes highlights several significant trends in the average relative flows. For the H_1_ drip irrigation tape, under different fertilizer concentration treatments, the highest average relative flow was observed in the E_1_ treatment (0.670), which was significantly higher than both E_2_ and E_3_ treatments. This indicates that higher fertilizer concentrations, like in the E_1_ treatment, may enhance the flow stability in this particular tape. Similarly, the sediment content in the H_1_ drip irrigation tape had a pronounced effect on the flow, with the F_1_ treatment showing the highest average relative flow (0.699). This suggests that lower sediment content positively impacts flow performance, as all comparisons between F_1_, F_2_, and F_3_ were statistically significant. In the H_2_ drip irrigation tape, the results were somewhat different. While the E_1_ treatment again produced the highest flow (0.618), the difference between the E_2_ and E_3_ treatments was not significant. This could imply that H_2_ tapes are less sensitive to variations in fertilizer concentration beyond a certain point. Regarding sediment content, the F_1_ treatment consistently showed a higher average relative flow (0.681) than the other sediment content treatments, reinforcing the idea that cleaner water conditions improve irrigation efficiency in these systems. For the H_3_ drip irrigation tape, the average relative flow of the F_1_ treatment was highest (0.698), and there was a significant 12.1% decrease in flow from F_1_ to F_3_. This pattern, observed across all treatments, suggests a strong inverse relationship between sediment content and flow performance in the H_3_ tape. Additionally, in terms of fertilizer concentration, the E_1_ treatment (0.668) had the highest relative flow, with significant differences noted across all treatments, indicating that both fertilizer concentration and sediment content substantially influence the performance of the H_3_ tape.

### 3.5. Analysis of influences of different working conditions on average relative flow

**Fig 5:**
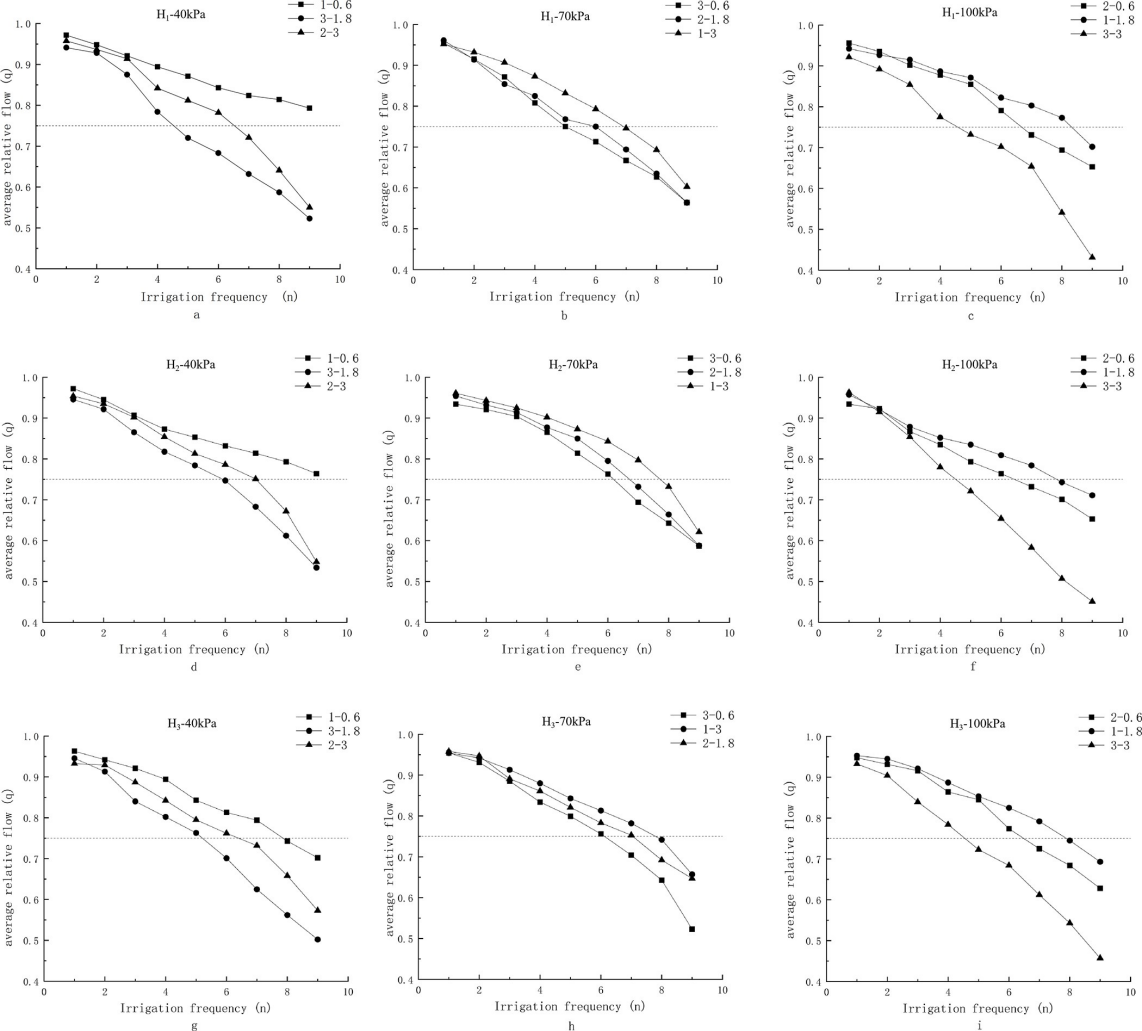
Changes in average relative flow under different conditions. Note: (a)–(c) show the average relative flow rate of H_1_-type drip irrigation tape under working pressures of 40 kPa, 70 kPa, and 100 kPa, respectively, under different sediment content and fertilizer concentration conditions. (d)–(f) show the performance of H_2_-type drip irrigation tape under the same pressure conditions, while (g)–(i) display the performance of H_3_-type drip irrigation tape. (’1–0.6’ represents a sediment content of 1 g/L and a fertilizer concentration of 0.6 g/L).

It can be seen from the previous analysis that the working pressure had no significant effect on the clogging of the dripper in muddy-water condition. Therefore, only the effects of the sediment concentration and fertilizer concentration on the clogging of the dripper are discussed in this section. Fig 5(A)–5(I) show the average relative flow of the H_1_-type, H_2_-type, and H_3_-type drip irrigation tapes under different working pressures of 40, 70, and 100 kPa, respectively. The notation "1–0.6" represents working conditions with a sediment content of 1 g/L and a fertilizer concentration of 0.6 g/L.

By comparing Fig 5(A)–5(C), it can be seen that the relative flow of the integrated irrigation with fertilizer irrigation decreased with the increase in the irrigation frequency under muddy-water conditions. With the increase in the irrigation times, the relative flow decreased more rapidly, the clogging of the dripper occurred faster, which inevitably meant that the fertilization in muddy water would significantly accelerate the clogging of the drippers. The comparison of Fig 5(A)–5(C) shows that with a fixed sediment content of 1 g/L, when the working pressure was 40 kPa, the dripper still had not been clogged after the 9^th^ irrigation process, while when the working pressure was 70 and 100 kPa, the dripper clogged after 7^th^ and 9^th^ irrigation processes, respectively. This indicated that the working pressure in muddy-water fertilization had no significant effect on the clogging of the dripper, which was consistent with the above variance analysis results. When the fertilizer concentration was 3 g/L, after the 9^th^ irrigation process, the clogging degree of the drip irrigation tape was significantly greater than those at concentrations of 1.8 and 0.6 g/L. The degree of clogging was in the order of 3.0 g/L > 1.8 g/L > 0.6 g/L. It can be concluded that the concentration of chemical fertilizer increased the degree of clogging to a certain extent.

The application and dissolution of chemical fertilizers introduce a large number of cations into the irrigation water. These cations, including Ca^2+^, Mg^2+^, Na^+^, and K^+^, neutralize the negatively charged surfaces of fine-grained sediments, compressing the double layer structure on the sediment surface. This reduction in electrostatic repulsion enhances inter-particle bonding and promotes the flocculation effect, which increases the likelihood of emitter clogging. Additionally, phosphate fertilizers can promote carbonate precipitation by reacting with calcium ions, leading to the formation of calcium phosphate. This process reduces the availability of free calcium, shifting the balance toward calcium carbonate precipitation, thereby further accelerating emitter clogging [20]. As shown in Fig 5(A)–5(C), under a working pressure of 40 kPa, the average relative flow of the three groups—1–0.6, 3–1.8, and 2–3—was 0.6, 1.8, and 3.0, respectively. The combination 1–0.6, representing lower sediment and fertilizer concentrations, experienced less flow reduction compared to groups 3–1.8 and 2–3, which had either higher sediment or fertilizer concentrations. In Fig 5(A), the flow reduction in group 1–0.6 was notably lower than in the other two groups, while the flow reduction for groups 2–3 and 3–1.8 was relatively similar. These results confirm that sediment concentration has a more pronounced impact on clogging than fertilizer concentration. Specifically, the higher the sediment concentration, the greater the flow reduction, indicating that sediment exerts a stronger influence on the average relative flow.

It can be seen from Fig 5(B) that the three combinations resulted in similar variations in the average flow. Therefore, under continuous irrigation conditions, there were strong similarities in the flow and the achieved average relative flows between the three groups. It can be seen that dripper blockage was not a single physical blockage or chemical blockage in the process of muddy-water fertilization and irrigation, but a joint effect and mutual promotion occurred to caused the blockage, which was consistent with the results of previous studies [21, 22]. In conclusion, in the process of fertilization and irrigation with muddy water, the clogging of the dripper is not controlled by single physical or chemical factors, but the result of the comprehensive effect of both physical and chemical factors, which is consistent with the findings of previous studies. As shown in Fig 5, the type-H_1_, type-H_2_, and type-H_3_ drip irrigation tapes shared the same clogging pattern under the same conditions. This indicates that the same labyrinth channel exhibits a similar clogging pattern at different flow.

## 4. Discussion

At the 95% confidence level, the variance analysis results showed that the influence of the working pressure on the average relative flows of the three drip irrigation tapes did not reach significant levels. This indicated that the working pressure had no significant effect on the average relative flow of the dripper under the test conditions. However, Liu et al. studied the effect of different working pressure levels on the clogging of the dripper by using water with a high- sediment content and found that the anti-clogging ability of the dripper gradually decreased as the working pressure dropped from 100 to 40 kPa or even lower [23]. Some researchers have suggested that under high-sediment-content irrigation conditions, the sediment is more likely to settle in the pipeline, thereby reducing the flow or clogging the dripper [24, 25]. This is mainly because different influences were considered than in this study. It is necessary to consider high sediment content in subsequent studies.

The results of this experiment suggest that the sediment content had a direct impact on the clogging of the dripper. In a muddy-water irrigation process, sediment filtration and other treatments before irrigation cannot completely eliminate solid particles in the irrigation water, and sediment particles entering the dripper flow channel with particle sizes less than 0.1 mm will still cause physical clogging [26]. Because the number of fine sediment particles in muddy water is relatively high, when the fine sediment particles enter the drip irrigation channel through the filter, the particles will aggregate to form larger-sized sediment particles, which will gradually accumulate and eventually clog the dripper [27]. The larger the particle size and the higher the concentration of sediment particles are in the irrigation water, the higher the sedimentation risk factor of the sediment particles is, and the more likely it is to result in the clogging of the dripper [28]. This experiment also found that under the same irrigation pressure and fertilizer concentration conditions, when the sediment content was 1 g/L, the clogging occurred after the 9^th^ irrigation process. When the sediment content was 2 g/L, the clogging occurred after the 7^th^ irrigation process. When the sediment content was 3 g/L, the clogging occurred after the 6^th^ irrigation process. To prevent the clogging of the drippers, when the sediment content in the irrigation water is high, the irrigation frequency should be appropriately reduced. This is consistent with the results of Wei et al. Their study suggested that when using particles with diameters less than 0.1 mm, a certain concentration of sediment is a necessary condition for particle collision and flocculation [29]. With the increase in the sediment concentration, the distribution of the particle concentration in the flow channel of the dripper increased as well. When the sediment concentration reached a certain level (1.25 g/L), the particle concentration had a significant effect on the clogging of the dripper.

The test results demonstrate that the concentration of chemical fertilizer had a direct impact on the clogging of the dripper. Within a certain range, as the fertilization concentration increased, the clogging of the dripper was more evident. This finding was consistent with the results of the study conducted by Zhou et al. [30]. This is because most fine-grained sediments are negatively charged, and cations can neutralize the compressed sediments. Application of fertilizer has been shown to alter the drip irrigation water source parameters, such as the type and concentration of nutrients, number of suspended particles, temperature, pH, and electrical conductivity. This causes mutual collision, adsorption, agglomeration, and precipitation of various solutes in the flow channel, generates turbulent flow, and leads to changes in the clogging and deposition process, thereby increasing the risk of clogging in the dripper [31]. Fertilization will also change the concentration of cations in the water, and most fine-grained sediment particles are negatively charged. The cations neutralize and compress the electric by-layer structures on the particle surfaces of the sediment [32]. After fertilization, the flocculation of sediment is enhanced, and it is easier to form a stable agglomerated structure to clog the dripper [33]. Zhou et al. pointed out that the flocculation sedimentation and structure are both affected by the ion concentration [34]. In integrated irrigation with fertilizer, the fertilizer will introduce a large quantity of cations, which will strengthen the flocculation between sediment particles [35]. Duffadar et al. pointed out that the increase in the cation concentration will enhance the bonding forces between particles, accelerate particle aggregation, and aggravate flocculation, resulting in clogging of the dripper [36].

The clogging sensitivity to sediment concentration and fertilizer concentration is different, and there is a complex coupling effect between these two factors. The concentration of chemical fertilizer affects dripper clogging by altering the physical, chemical, and biological characteristics of solutes in water, highlighting the need for further exploration of how fertilizer type and concentration influence physical, chemical, and biological clogging. Due to multiple practical factors, the types of fertilizers and drip irrigation tape samples used in this study are limited. More fertilizer types and drip tape types will be considered in future experiments. Also, it is necessary to further explore the inducement mechanism of dripper clogging in drip irrigation system by means of the content of clog, the total number of microorganisms and sediment adsorption.

## 5. Conclusions

This study explores the combined effects of fertilizer concentration, sediment content, and working pressure on the clogging performance of single-wing labyrinth drip irrigation tapes under muddy-water conditions. The findings offer important insights into the critical factors affecting dripper performance and suggest practical strategies for improving drip irrigation system design.

Firstly, the study demonstrates that sediment content and fertilizer concentration are the primary factors contributing to dripper clogging, while working pressure plays a comparatively minor role. Across all tested drip tapes (H_1_, H_2_, and H_3_), the highest average relative flows were observed at lower sediment content (1g/L) and fertilizer concentration (0.6g/L), underscoring the importance of managing these variables to minimize clogging risks during muddy-water irrigation.

Secondly, variance analysis reveals that both sediment content and fertilizer concentration significantly impact the average relative flow, with higher levels of both factors leading to more severe clogging. This aligns with prior research, which indicates that interactions between sediment particles and fertilizer components promote chemical precipitation and particle aggregation, thereby exacerbating dripper clogging.

Lastly, the results have significant implications for the design and management of integrated water-fertilizer irrigation systems. It is recommended to reduce irrigation frequency and employ sediment filtration techniques to mitigate clogging under high-sediment conditions. Future research should examine the long-term effects of various combinations of fertilizers and sediments on dripper performance to optimize the anti-clogging properties of drip irrigation systems.

In conclusion, this research highlights the essential role of sediment and fertilizer management in maintaining drip irrigation efficiency and provides a foundation for future studies aimed at sustainable irrigation practices.

## Supporting information

S1 Graphical abstract (JPG)
