# Supplementary figures and images for: Analysis of clogging factors in single-wing labyrinth drip irrigation tape
Source: PLoS One. 2024 Dec 31;19(12):e0313888. doi: 10.1371/journal.pone.0313888 (PMC11687791; doi:10.1371/journal.pone.0313888)

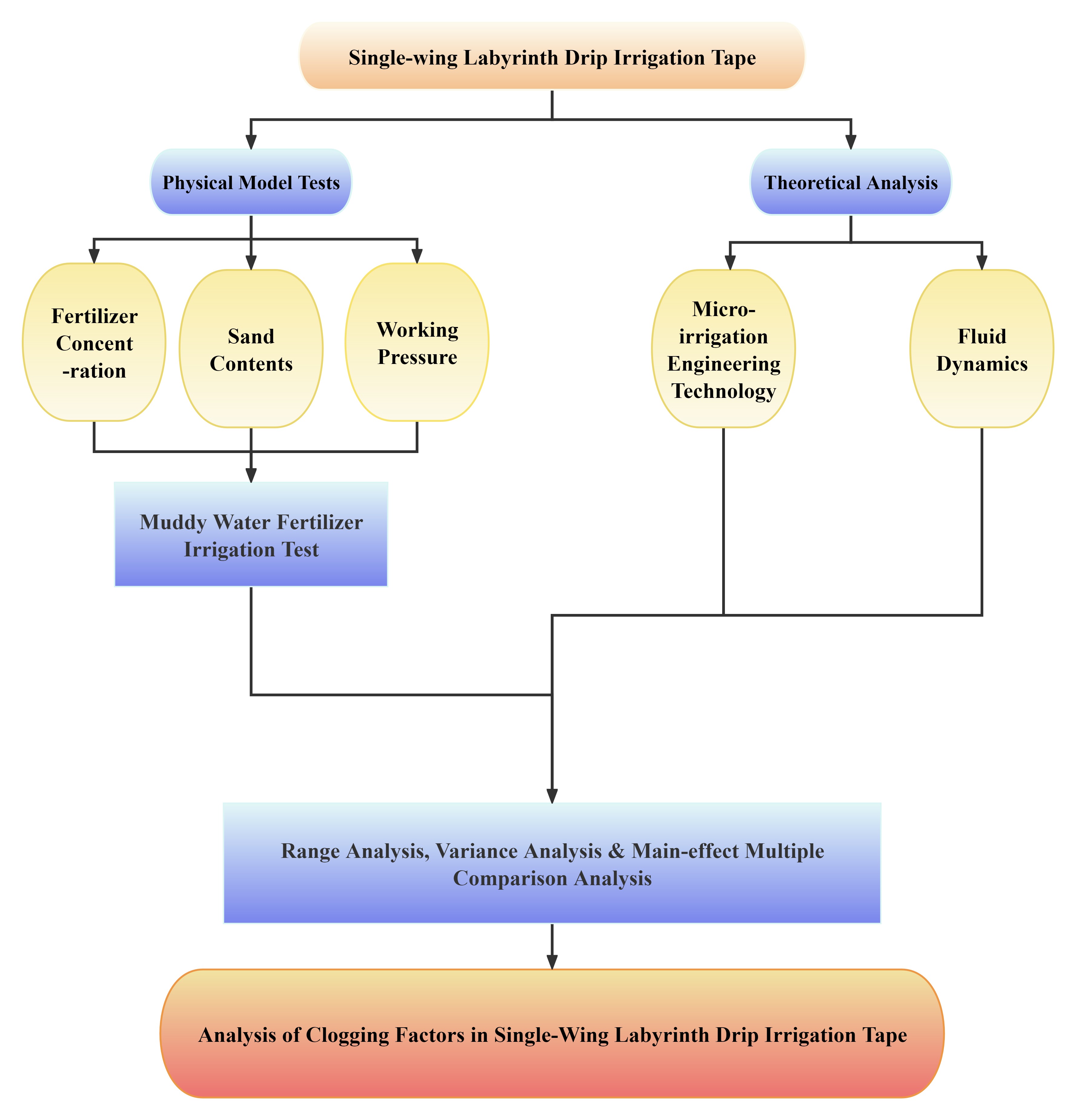

Supplement: S1 Graphical abstract — (JPG) [file pone.0313888.s001.jpg]
